# The dental anxiety scale (DAS) – psychometric properties and longitudinal findings among middle-aged adults

**DOI:** 10.1186/s40359-025-03304-9

**Published:** 2025-08-21

**Authors:** David Bantel, Witold X. Chmielewski, Elmar Brähler, Yve Stöbel-Richter, Markus Zenger, Katharina Marilena Weil, Hendrik Berth

**Affiliations:** 1 Carl Gustav Carus Faculty of Medicine, Division of Psychological and Social Medicine and Developmental Neuroscience, Research Group Applied Medical Psychology and Medical Sociology, Technische Universität Dresden, Fetscherstr. 74, 01307 Dresden, Germany; 2 Institute for Psychological Psychotherapy, University of Bochum, Bochum, Germany; 3 Department of Psychosomatic Medicine and Psychotherapy, University Medical Center of the Johannes Gutenberg-University, Mainz, Germany; 4 Department of Medical Psychology and Medical Sociology, University Hospital Leipzig, Leipzig, Germany; 5 Faculty of Managerial and Cultural Studies, University of Applied Sciences Zittau/Görlitz, Görlitz, Germany; 6 Department of Applied Human Studies, University of Applied Sciences Magdeburg-Stendal, Stendal, Germany; 7 Integrated Research and Treatment Center Adiposity Diseases—Behavioral Medicine, Psychosomatic Medicine and Psychotherapy, University Medical Center of the University, Leipzig, Germany

**Keywords:** Dental anxiety scale, DAS, Longitudinal study, Oral health, Adults

## Abstract

**Background:**

The Dental Anxiety Scale (DAS) is a well-established instrument for assessing dental anxiety. While numerous cross-sectional studies have examined the DAS, longitudinal research investigating age-related changes, reliability, validity over time, associations with mental and physical health, and demographic determinants remains limited. Furthermore, the possibility of longitudinal changes in its factor structure has received limited attention.

**Methods:**

In 2013/2014, *N* = 329 German adults (53.6% female, mean age 40.20 years) and in 2019/2020, *N* = 323 adults (55.7% female, mean age 47.15 years) completed questionnaires, including the DAS, Oslo Social Support Scale (OSSS-3), Health-Score (G-Score), Patient Health Questionnaire (PHQ-2), Generalized Anxiety Disorder Screener (GAD-2), Short Scale for General Life Satisfaction (L-1), and oral health behavior items. DAS reliability was assessed using Cronbach’s alpha and McDonald’s omega, and factor structure via principal component analyses (PCA). Longitudinal dynamics were analyzed using McNemar’s test. Associations between dental anxiety and independent variables were investigated using correlational analyses and Wilcoxon signed-rank tests.

**Results:**

DAS significantly declined from 2013/2014 (M = 9.47) to 2019/2020 (M = 9.21). Internal consistency was excellent (α/Ω = 0.93), and a correlation of dental anxiety across waves was high (rs = 0.79). PCA revealed a stable one-factor structure (2013/2014: 82.25%, 2019/2020: 82.86% explained variance). McNemar test indicated no significant changes in the proportion of subjects with or without dental anxiety over time. Higher dental anxiety was significantly correlated with lower levels of: life satisfaction, perceived influence over health, condition of health, physical and mental health, and social support in both waves (all rs ≥ 0.14; *p* ≤ 0.015). A significant decrease in dental anxiety over time was observed in participants with frequent tooth brushing, no indication for depression, and at least moderate social support (all z ≤ -2.07, all *p* ≤ 0.019).

**Conclusions:**

This study provides evidence for DAS’s reliability, temporal stability, and construct validity. Although dental anxiety showed a statistically significant decline it remains relatively stable for most participants. Findings indicate meaningful associations with psychosocial, behavioral, and health-related factors, supporting the potential value of interdisciplinary approaches that include psychological support in oral health care.

## Background

Dental anxiety is defined as an intense emotional response to elements of the dental treatment situation that causes distress for affected individuals and appears exaggerated in relation to the actual risks present [1–3]. It is associated with the anticipation or experience of invasive procedures and manifests in psychological (e.g., feelings of threat and discomfort, specific cognitive distortions), somatic (e.g., physiological reactions such as muscle tension, tremor), and behavioral responses [4–6]. Affected individuals often delay or avoid necessary care - including both oral health behaviors (OHB) and professional treatment - which can lead to a deterioration in oral health and ultimately necessitate more complex and potentially anxiety-provoking interventions [5, 7–9]. This vicious cycle presents challenges for both the patient and the oral health professional, leading to diminished compliance, extended treatment durations, and a generally more adverse clinical environment [10–12]. Dental anxiety has a high global prevalence across all age groups [13–15], with estimates between 5 and 30% in the general population [3, 16, 17]. It not only compromises individual well-being but also places a significant burden on healthcare systems by increasing the demand for extensive and costly interventions, thereby raising overall healthcare expenditures [8–10, 18].

Dental anxiety has been the subject of scientific research for several decades, with pioneering studies emerging in the 1950s [19, 20]. Since then, several well-established self-report instruments have been developed for its assessment, including Corah’s Dental Anxiety Scale (DAS; [1]), Kleinknecht’s Dental Fear Survey (DFS; [21]), Humphris’s Modified Dental Anxiety Scale (MDAS; [22]), and Armfield’s Index of Dental Anxiety and Fear (IDAF-4 C; [23]). As the oldest of the instruments mentioned, the DAS has been widely employed in international research and continues to serve as a reference measure in many studies worldwide [1, 2, 8, 24–27]. Due to its strong psychometric properties and conciseness, it proved to be a practical and cost-effective tool to include into large population-based studies with minimal burden on participants or researchers [3, 8, 24, 25]. However, more recent instruments offer important advantages - for example, the MDAS includes an additional item on fear of local anesthesia (i.e. injection), a clinically relevant component [22], and the IDAF-4 C is grounded in a clear theoretical framework and distinguishes between emotional, cognitive, behavioral, and physiological components of dental anxiety [23]. In the present study, the DAS was selected due to the availability of comparable data from a previous wave of the Saxony Longitudinal Study (SLS), thereby enabling consistent and meaningful longitudinal comparisons.

Most studies utilizing the DAS rely on cross-sectional designs, providing valuable insights into the prevalence and correlates of dental anxiety at a single point in time [1, 2, 12, 24–27]. Comparatively few studies, however, have employed a longitudinal design - an approach that is essential for understanding the development, persistence, and long-term consequences of dental anxiety within specific populations [16, 17, 28–32]. While previous research on younger adults has been somewhat inconclusive regarding age-related changes in dental anxiety [32–34], and studies in middle-aged and older adults tend to suggest a decline with increasing age [29, 30, 34], it remains unclear whether age-related changes are also reflected in the underlying factor structure of the DAS. Moreover, the long-term influence of additional contributing factors identified in cross-sectional studies - such as oral health behaviors, mental and physical health or social determinants - remains insufficiently understood and warrants further investigation. While dental anxiety has consistently been shown to impact both oral health care and oral health, fewer studies have explored its associations with mental and physical health, as well as with demographic and social determinants in a longitudinal setting [28, 35–38]. Regarding mental health, dental anxiety has been associated with various psychiatric conditions, including comorbid phobias, anxiety disorders, and depression, as well as with parallel changes in dental anxiety and mental health over time [39, 40]. For instance, findings from a Finnish population-based survey indicated that individuals reporting pronounced dental anxiety also showed a higher prevalence of depressive and anxiety disorders compared to those with little or no fear [34]. Likewise, a longitudinal study from New Zealand demonstrated that young adults with higher levels of dental anxiety were more likely to exhibit mental disorders, which appeared to play a role in the continuation of dental anxiety over time [33]. Dental anxiety has also been associated with poor physical health, lower health-related self-efficacy, and reduced quality of life [27, 41, 42] including more specifically oral health-related quality of life (OHRQoL; [25, 43, 44]). However, the extent to which physical health status and health-related expectations influence dental anxiety in a longitudinal context remains insufficiently understood. Although demographic and social factors such as sex, relationship status, parenthood, and social integration have repeatedly been linked to dental anxiety, social determinants remain understudied in longitudinal research [42, 45–47].

Additionally, despite its widespread use, the DAS has not been sufficiently validated in culturally specific contexts - an essential step in accounting for regional differences in dental anxiety. To our best knowledge, no validation study with a longitudinal design has yet been conducted with a Central European, and more specifically, German sample. Consequently, the present study aimed to investigate the long-term psychometric properties of the DAS in a German sample. This included examining the longitudinal associations between dental anxiety, physical and mental health, oral health behaviors, and other variables as well as exploring potential changes in the scale’s underlying factor structure over time. Accordingly, the present study pursued three primary objectives:

First, it aimed to examine longitudinal changes in dental anxiety over a seven-year period within a cohort of aging adults.

Second, it sought to evaluate the factor structure and internal consistency of the Dental Anxiety Scale (DAS) at two measurement points (2013 and 2020).

Third, it investigated the associations between dental anxiety and a range of psychosocial, behavioral, and health-related variables.

Collectively, these objectives are intended to contribute to a more comprehensive understanding of the stability and correlates of dental anxiety in mid- to later adulthood.

## Materials and methods

### Study design and recruitment of participants

This study is part of the Saxon Longitudinal Study (SLS; [48, 49]), a long-term research initiative originally launched by the Central Institute for Youth Research of the former German Democratic Republic (GDR). Since 1987, the same cohort has participated in the study. Each year, they completed a comprehensive set of questionnaires. The first wave included 1,407 predominantly 14-year-old students from randomly selected schools from Leipzig and Chemnitz districts, forming a gender-balanced, age-homogeneous sample representative of the 1973 East German cohort. After secondary school and the planned end of the study in 1989 (*N* = 1,281), 587 participants agreed to continue in later waves.

The data analyzed in this study is drawn from two waves. 27th wave took place from November 2013 to March 2014; 31st wave was conducted from November 2019 to March 2020. After controlling for missing data, a total of 287 subjects participated in both waves (329 in 2013/2014 and 323 in 2019/2020). In certain analyses, the number of cases is slightly reduced due to missing values in specific items and/or scales. The study materials were distributed to participants via mail and were returned through the same method. This research was conducted in accordance with the STROBE guidelines [50].

### Questionnaires

#### Dental anxiety scale (DAS)

Corah’s Dental Anxiety Scale (DAS; [1, 51]) is an internationally recognized self-report questionnaire. It consists of only four items, each assessing anxiety levels in specific treatment situations. Participants respond on a five-point Likert scale, ranging from 1 (relaxed) to 5 (highly anxious). The items are:

1. *“If you had to go to the dentist tomorrow*,* how would you feel about it?”*
2. *“When you are waiting in the dentist’s office for your turn in the chair*,* how do you feel?”*
3. *“When you are in the dentist’s chair waiting while he gets the drill ready to begin working on your teeth*,* how do you feel?”*
4. *“You are in the dentist’s chair to get your teeth cleaned. While you are waiting and the dentist is getting out the instrument which he will use to scrape your teeth around the gums*,* how do you feel?”*

The total score ranges from 4 to 20, with a general population mean of 8. A cut-off score of 15 differentiates individuals with severe dental anxiety (≥ 15), moderate dental anxiety ( [13]– [14]), and low to no dental anxiety (≤ 12). The validity of the DAS has been confirmed in multiple studies [1, 2, 12]), demonstrating strong psychometric properties, including good to excellent reliability (rtt = 0.86 [1] and 0.94 [2]) and good to excellent internal consistency, with Cronbach’s alpha ranging from 0.86 to 0.93 [1, 8, 27].

### Oslo social support scale (OSSS-3)

The level of social support was investigated using the internationally recognized Oslo Social Support Scale (OSSS-3; [52, 53]). Due to its brevity and economic properties, this self-report questionnaire is recommended for population-based research. The OSSS-3 consists of three items, each requiring participants to select the most appropriate response, for example:

1. *“How many people are so close to you that you can count on them if you have great personal problems?”*

- Response options: 1 = “*none”*, 2 = “*one or two”*, 3 = “*three to five”*, 4 = “*more than five”*.

0. *“How much interest and concern do people show in what you do?”*

- Response options: 1 = “*none”*, 2 = “*little”*, 3 = “*uncertain”*, 4 = “*some”*, 5 *= “a lot”*.

0. *“How easy is it to get practical help from neighbors if you should need it?”*

- Response options: 1 = “*very difficult”*, 2 = “*difficult”*, 3 = “*possible”*, 4 = “*easy”*, 5 *= “very easy”*.

The total score ranges from 3 to 14, allowing for the categorization of participants into high [12–14], moderate [9–11], and low [3–8] levels of social support. The validity of the OSSS-3 has been demonstrated in various studies [53], confirming its adequate psychometric properties, including acceptable reliability (rtt = 0.62) and acceptable internal consistency, with Cronbach’s alpha ranging from 0.63 to 0.70 [54].

### Health-score (G-score)

The G-Score (“Gesundheitsscore”) was used to assess participants’ subjectively perceived physical health over the past 12 months [44]. The self-report questionnaire is shorter than other instruments measuring similar constructs [55, 56] and was particularly developed for the Saxon Longitudinal Study. Participants received the following instructions: *“Have you experienced any of the following complaints in the past 12 months? Please indicate how often they occurred.”* The questionnaire consists of four items:

1. *Nervousness*
2. *Stomach discomfort*
3. *Sleeping difficulties*
4. *Heart-related complaints*.

Responses are recorded on a four-point scale: 0 = “*no*,* never”*, 1 = “*yes*,* rarely”*, 2 = “*yes*,* occasionally”*, 3 = “*yes*,* frequently”*. The total score ranges from 0 to 12, with higher values indicating a greater burden of physical complaints. Due to its long-term application in the Saxon Longitudinal Study, a comprehensive database is available for reference [49]. The validity of the G-Score has been confirmed previously, including acceptable reliability (rtt = 0.66) and acceptable internal consistency (Cronbach’s alpha = 0.66) [57].

### Patient health questionnaire-2 (PHQ-2) and generalized anxiety disorder screener (GAD-2)

The PHQ-2 [58] and the GAD-2 [59] represent the two subscales of the well-established 4-item self-report questionnaire PHQ-4 [60]. The two PHQ-2 items assess the core diagnostic criteria for depressive disorders, and the two GAD-2 items assess the core diagnostic criteria for the generalized anxiety disorder, as outlined in the Diagnostic and Statistical Manual of Mental Disorders, 4th Edition (DSM-IV; [61]). Each item represents a distinct symptom. Following the structure of the original scale, PHQ-2 and GAD-2 both begin with the prompt: *“Over the last two weeks*,* how often have you been bothered by the following problems?”* followed by the respective items for:

### PHQ-2

1. *“Little interest or pleasure in doing things”*.
2. *“Feeling down*,* depressed*,* or hopeless”*.

### GAD-2

0. *“Feeling nervous*,* anxious or on edge"*.
0. *“Not being able to stop or control worrying”*.

Responses are rated on a 4-point scale ranging from 0 (*“not at all”*) to 3 (*“nearly every day”*), resulting in a total score for each subscale ranging from 0 to 6. A cut-off score of 3 or higher on either the PHQ-2 or GAD-2 is recommended for identifying individuals at risk for depression or anxiety, respectively. The combination of the subscales (i.e. PHQ-4) has demonstrated solid psychometric properties, with acceptable internal consistency (Cronbach’s alpha = 0.85) and factorial validity, supported by exploratory and confirmatory factor analyses confirming its two-factor structure (60).

### Short scale for general life satisfaction (L-1)

The Short Scale for General Life Satisfaction L-1 (“Kurzskala zur Erfassung der Allgemeinen Lebenszufriedenheit“) was used to assess participants’ general life satisfaction [62]. The self-report questionnaire consists of only one item and asks: “*All things considered*,* how satisfied are you with your life these days?”*, rated on a four-point scale from 1 (“dissatisfied”) to 5 (“satisfied”). The validity of the L-1 has been demonstrated previously, confirming its adequate psychometric properties, including acceptable reliability (rtt = 0.67 [62]).

### Oral health behaviors (OHB) and other health variables

OHB were assessed using two specifically designed questions:

1. *“How often do you go to the dentist each year?”*

- Response options: 1 = “*never”*, 2 = “*once a year”*, 3 = “*twice a year”*, 4 = “*more than twice a year”*.

0. *“How frequently do you brush your teeth daily?”*

- Response options: 1 = “*never”*,* 2 = “once a day”*,* 3 = “twice a day”*,* 4 = “three times a day”*,* 5 = “four times a day”*,* 6 = “more than four times a day”*.

Participants also rated their perceived condition of health by responding to the following question, adapted from the German Federal Health Survey (Bundesgesundheitssurvey; [63]):

1. *“How would you rate your current health status?“*,

- Response options: 1 = “*poor”*,* 2 = “fair”*,* 3 = “satisfactory”*,* 4 = “good”*,* 5 = “very good”*.

In addition, levels of perceived influence over health were measured with the question:

0. *“What is your opinion on how much you can influence your own status of health?”*

- Response options: 1 = “*not at all”*,* 2 = “little”*,* 3 = “to some extent”*,* 4 = “a lot”*,* 5 = “very much”*.

### Statistical analysis

Statistical analyses were conducted using SPSS 28 (IBM). Descriptive statistics including mean (M) and standard error of the mean (SEM) were reported. Normality of the data was tested using the Shapiro-Wilk test. Equality of variances across groups was assessed with Levene’s test. The reliability of the Dental Anxiety Scale (DAS) was evaluated through Cronbach’s alpha and McDonald’s omega to measure internal consistency. Factor structure was examined by conducting Principal Component Analysis (PCA) with varimax rotation for both waves. Wilcoxon signed-rank tests (with median, iqr: inter quartile range, z-value) were applied to compare dental anxiety across waves when data were non-normally distributed. Statistical significance was determined at a *p*-value of less than 0.05. The McNemar test was employed to compare the proportion of participants with varying levels (severe, moderate, low to no) of dental anxiety across the two waves. Correlation analyses (Spearman’s Rho) were conducted in both waves to explore and compare variables associated with dental anxiety. Further comparisons of dental anxiety, based on demographic factors (sex, age, partnership), oral health behaviors (OHB), perceived condition of health, perceived influence over health, perceived physical health (G-Score), perceived mental health (PHQ-2, and GAD-2), and levels of life satisfaction (L-1), were performed using Wilcoxon signed-rank tests, where appropriate. To this end, subgroups were formed based on low versus high expression of relevant variables, e.g., no depression versus moderate to manifest depression according to the PHQ-2. Only participants who remained in the same category (e.g., no depression or moderate to manifest depression) across both waves were included. DAS values were then compared between wave 27 and wave 31 within these stable subgroups. The sample size required for robust statistical analysis was calculated with the G*power 3 software program [64], necessitating a minimum of 45 subjects [65] to achieve a predefined effect size of d = 0.05, a significance level of *p* = 0.05, and a power of 95% (1–ß = 0.95). This study was conducted in alignment with the Declaration of Helsinki [66] and received ethical approval from the Ethics Committee of the TUD Dresden University of Technology, Germany (EK EK8012011).

## Results

### Demographic characteristics

In wave 27 (in 2013/2014) of the Saxon Longitudinal Study (SLS), data from 329 participants (53.6% female; mean age 40.20 ± 0.03) were collected. In wave 31 (in 2019/2020), 323 participants were included (55.7% female; mean age 47.15 ± 0.03). A total of 287 subjects participated in both waves. Key sociodemographic characteristics did not differ significantly between participants of both waves and those of only one wave (not shown in detail here). Further descriptive data are presented in Table 1.

**Table 1:** Demographic data for both waves

Demographic Data in (% or mean ± SEM)	Sex	Age	Partner	Number of children	DAS	DAS no/low anxiety	DAS moderate anxiety	DAS severe anxiety	L-1	Annual dentist appointments	Frequency of tooth brushing	G-Score	Perceived condition of health	Perceived influence over health	PHQ-2	GAD-2	OSSS-3
**Wave 27**: **2013/2014** (***N*** **= 329)**	53.6% ♀	40.20 ± 0.03	77.1% ± 0.03 yes	1.75 ± 0.05	9.47 ± 0.23	80.4%	8.0%	11.7%	2.25 ± 0.04	2.53 ± 0.04	2.93 ± 0.03	3.72 ± 0.15	2.34 ± 0.05	1.94 ± 0.05	2.88 ± 0.07	3.03 ± 0.07	10.28 ± 0.10
**Wave 31: 2019/2020** (***N*** **= 323)**	55.7% ♀	47.15 ± 0.03	81.4% ± 0.02 yes	1.79 ± 0.04	9.21 ± 0.23	81.4%	8.4%	10.2%	2.17 ± 0.45	2.59 ± 0.04	2.91 ± 0.03	3.77 ± 0.16	2.43 ± 0.05	1.93 ± 0.04	2.85 ± 0.06	2.92 ± 0.07	10.52 ± 0.11

Demographic data for sex (female ♀), age (in years), partnership (yes/no), number of children, dental anxiety measured with DAS (≤ 12: no/low anxiety, 13–14: moderate anxiety, ≥15: severe anxiety), life satisfaction measured with L-1 (1: dissatisfied − 5: satisfied), annual dentist appointments (1: never − 4: more than twice), daily frequency of tooth brushing (1: never − 6: more often than four times), perceived physical health measured with G-Score (complaints regarding nervousness, stomach complaints, insomnia, and heart complaints 0: never − 3: frequently), perceived condition of health (1: poor − 5: very good), perceived influence over health (1: not at all − 5: a great deal), indication for depression measured with PHQ-2 (0: not at all − 3: nearly every day), indication for anxiety measured with GAD-2 (0: not at all − 3: nearly every day), and social support measured with OSSS-3 (1: none − 4: more than five). % or mean ± SEM are reported.

### Internal consistency

The internal consistency of the Dental Anxiety Scale (DAS) in this study was confirmed to be excellent, with Cronbach’s alpha reaching 0.93 in both wave 27 and wave 31. Similarly, McDonald’s omega also indicated excellent reliability with a score of 0.93 in each wave. Detailed statistical analyses including means, Standard Errors of Measurement (SEMs), skewness, kurtosis, alpha if item deleted, and corrected item-total correlations are provided in Table 2.

**Table 2:** Internal consistency for both waves

Internal consistency	DAS Item 1	DAS Item 2	DAS Item 3	DAS Item 4	Cronbach’s Alpha	McDonald’s Omega
**Wave 27: 2013/2014 (** ***N*** ** = 329)**					0.93	0.93
- Mean ± SEM	2.64 ± 0.06	2.03 ± 0.06	2.63 ± 0.06	2.23 ± 0.06		
- Skewness	0.80	0.98	0.53	0.70		
- Kurtosis	0.15	0.36	-0.32	-0.07		
- Alpha if Item deleted	0.92	0.90	0.89	0.91		
- Corrected item total correlation	0.80	0.86	0.87	0.81		
**Wave 31: 2019/2020 (** ***N*** ** = 323)**					0.93	0.93
- Mean ± SEM	2.57 ± 0.06	2.00 ± 0.06	2.56 ± 0.06	2.16 ± 0.06		
- Skewness	0.95	1.04	0.58	0.79		
- Kurtosis	0.47	0.55	-0.29	>-0.01		
- Alpha if Item deleted	0.92	0.90	0.90	0.92		
- Corrected item total correlation	0.80	0.88	0.87	080		

Internal consistency for DAS items 1–4 (1. “If you had to go to the dentist tomorrow, how would you feel about it?“, 2. “When you are waiting in the dentist’s office for your turn in the chair, how do you feel?“, 3: “When you are in the dentist’s chair waiting while he gets the drill ready to begin working on your teeth, how do you feel? And 4. “You are in the dentist’s chair to get your teeth cleaned. While you are waiting and the dentist is getting out the instrument which he will use to scrape your teeth around the gums, how do you feel?”): in waves 27 (2013/2014) and 31 (2019/2020). Mean ± SEM, skewness, kurtosis, alpha if item deleted and corrected item total correlation for DAS item 1–4 are reported. Cronhach’s Alpha and McDonald’s Omega are reported for the DAS-Score.

Internal consistency was also assessed for the additional questionnaires used. For the OSSS-3, internal consistency was found to be poor, with Cronbach’s alpha values of 0.53 (2013) and 0.48 (2020), and McDonald’s omega values of 0.54 (2013) and 0.49 (2020). The G-Score showed acceptable reliability, with Cronbach’s alpha values of 0.72 (2013) and 0.76 (2020), and McDonald’s omega values of 0.73 (2013) and 0.77 (2020). The internal consistency of the PHQ-2 and GAD-2 was also acceptable across both waves. For the PHQ-2, Cronbach’s alpha was 0.78 in both 2013 and 2020. For the GAD-2, Cronbach’s alpha was 0.72 in 2013 and 0.78 in 2020. McDonald’s omega was not calculated for either scale due to the limited number of items.

### Longitudinal changes across waves

A highly significant strong correlation between DAS total scores in both waves was observed (rs = 0.79; *p* ≤ 0.001), suggesting that individual differences in dental anxiety remained relatively consistent over the seven-year period.

### Factor structure

To investigate the factor structure of the DAS, exploratory factor analyses were conducted in both waves of the study. Principal Component Analyses (PCA) with varimax rotation were utilized to understand the associations among the items of the instrument. The analyses were contingent upon meeting the prerequisite of substantial correlations among the items. This condition was satisfied, with correlations in wave 27 being at least 0.69 (all *p* ≤ 0.001), and in wave 31 being at least 0.66 (all *p* ≤ 0.001), as detailed in Table 3.

**Table 3:** Correlation analyses

	DAS Item 1	DAS Item 2	DAS Item 3	DAS Item 4
**Wave 27/Wave 31** (*N* = 326–329)				
DAS Item 1	1/1			
DAS Item 2	**0.78** ^***^ **/0.82** ^***^	1/1		
DAS Item 3	**0.75** ^***^ **/0.76** ^***^	**0.81** ^***^ **/0.83** ^***^	1/1	
DAS Item 4	**0.69** ^***^ **/0.66** ^***^	**0.74** ^***^ **/0.76** ^***^	**0.80** ^***^ **/0.80** ^***^	1/1

Correlation analyses for DAS items 1–4 (1. “If you had to go to the dentist tomorrow, how would you feel about it?“, 2. “When you are waiting in the dentist’s office for your turn in the chair, how do you feel?“, 3: “When you are in the dentist’s chair waiting while he gets the drill ready to begin working on your teeth, how do you feel?”, and 4. “You are in the dentist’s chair to get your teeth cleaned. While you are waiting and the dentist is getting out the instrument which he will use to scrape your teeth around the gums, how do you feel?”) in waves 27 (2013/2014) and 31 (2019/2020). Pearson’s r is reported. **Bold**: significant correlations, *italics*: trend-level significant correlations, ^***^ *p* < 0.001; ^**^ *p* < 0.01; ^*^ *p* < 0.05; ^(*)^ *p* < 0.10.

In both waves of the study, PCA yielded a one-factor solution. This result was supported by the scree plot, which consistently showed a distinct inflection point after the first factor. These findings indicate a stable factor structure of the DAS over time, supporting its continued applicability in an ageing sample. Specifically, in wave 27, the single extracted factor accounted for 82.25% of the variance (eigenvalue = 3.29). In wave 31, the factor explained 82.86% of the variance with an eigenvalue of 3.31. The factor loadings and communalities for all four items are presented in Table 4.

**Table 4:** Principal component analyses for both waves

DAS Items	DAS Item 1 factor loading on component 1 / communalities	DAS Item 2 factor loading on component 1 / communalities	DAS Item 3 factor loading on component 1 / communalities	DAS Item 4 factor loading on component 1 / communalities	Eigenvalue	% of variance
**Wave 27: 2013/2014** (*N* = 326)	0.89 0.79	0.92 0.85	0.93 0.86	0.89 0.79	3.29	82.25
**Wave 31: 2019/2020** (*N* = 315)	0.89 0.79	0.94 0.88	0.93 0.87	0.88 0.78	3.31	82.86

Principal component analysis for DAS items 1–4 (1. “If you had to go to the dentist tomorrow, how would you feel about it?“, 2. “When you are waiting in the dentist’s office for your turn in the chair, how do you feel?“, 3. “When you are in the dentist’s chair waiting while he gets the drill ready to begin working on your teeth, how do you feel?, 4. “You are in the dentist’s chair to get your teeth cleaned. While you are waiting and the dentist is getting out the instrument which he will use to scrape your teeth around the gums, how do you feel?”) in waves 27 (2013/2014) and 31 (2019/2020): Factor loadings on component 1, communalities, Eigenvalues and percentage of variance are reported.

### Comparison of dental anxiety across waves

A comparison of DAS total scores across waves revealed a modest, yet statistically significant decrease from wave 27 (9 [7–12]) to wave 31 (8 [6–11]; z = -1.75; *p* = 0.040). Among the 287 participants who took part in both waves, the majority (*n* = 213) exhibited neither moderate (i.e. DAS total score: 13–14) nor severe dental anxiety (i.e. DAS total score ≥ 15) at either time point, while 35 participants qualified for moderate to severe dental anxiety in both waves. Additionally, 18 participants who did not meet the criteria for moderate or severe dental anxiety in wave 27 developed it by wave 31, whereas 21 participants with moderate or severe dental anxiety in wave 27 no longer exhibited it in wave 31. The McNemar test - comparing the proportion of participants with no to low dental anxiety vs. the proportion of participants in a combined category of moderate to severe dental anxiety - indicated no significant difference (*p* < 0.749) between the proportion of these two groups over time. This suggests that there is no age-related shift in the prevalence of no to low vs. moderate to manifest dental anxiety over time. Dental anxiety (i.e. DAS total scores) also remained strongly correlated across the two waves (rs = 0.79; *p* ≤ 0.001), indicating a stable underlying pattern.

**Fig. 1:**
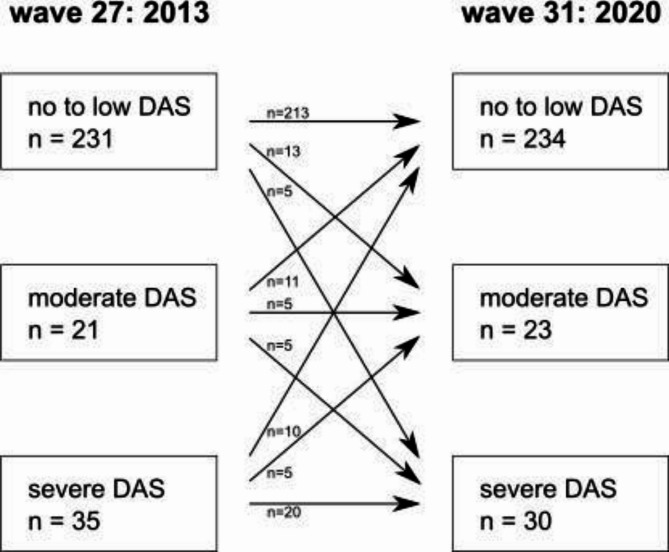
Stage transitions between trichotomized dental anxiety groups at two time points - wave 27 (2013) and wave 31 (2020) - among participants (*n* = 287) who completed the Dental Anxiety Scale (DAS) at both waves

A more detailed analysis of changes across waves, which distinguished between no to low, moderate, and severe dental anxiety, revealed notable transitions between categories, as shown in Fig. 1. Among the 231 participants with no to low dental anxiety in wave 27, 13 developed moderate, and 5 developed severe dental anxiety by wave 31. Of the 21 participants with moderate dental anxiety in wave 27, 11 shifted to no to low levels, while 5 progressed to severe dental anxiety. Among the 35 participants initially classified with severe dental anxiety, 10 improved to no to low, and 5 transitioned to moderate anxiety by wave 31. Although relatively stable for the majority, the findings indicate that dental anxiety is subject to dynamic changes over time, with both increases and decreases observed across categories.

### Correlation analyses (Spearman’s Rho)

In wave 27 (all rs ≥ 0.135; all *p* ≤ 0.015) and wave 31 (all rs ≥ 0.148; all *p* ≤ 0.008), higher levels of dental anxiety (i.e. DAS total scores) were significantly associated with lower levels of life satisfaction (L-1), lower levels of perceived physical health (G-Score), lower levels of perceived condition of health, lower levels of perceived influence over health, lower levels of perceived mental health (PHQ-2 and GAD-2), and lower levels of social support (OSSS-3) and vice versa. In wave 31, higher levels of dental anxiety were additionally associated on a trend-level with a lower number of dentist appointments (rs = -0.105; all *p* = 0.060). All correlations are depicted in Table 5.

**Table 5:** Correlation analyses based on DAS in wave 27 (2013/2014) and wave 31(2019/2020)

	DAS	L-1	Annual dentist appointments	Daily frequency of tooth brushing	G-Score	Perceived condition of health	Perceived influence over health	PHQ-2	GAD-2	OSSS-3
**Wave 27/31** (*N* = 322–329 / 313–323)										
DAS	1									
L-1	**0.16** ^**^ **/0.16** ^***^	1								
Annual dentist appointments	-0.09**/***-0.11*^(*)^	0.08/-0.08	1							
Daily frequency of tooth brushing	*0.09*^(*)^/0.04	0.03/-0.03	**0.11** ^*^ **/0.16** ^**^	1						
G-Score	**0.22** ^***^ **/0.26** ^***^	**0.30** ^***^ **/0.30** ^***^	0.15^**^/0.06	< 0.01/-0.05	1					
Perceived condition of health	**0.28** ^***^ **/0.28** ^***^	**0.28** ^***^ **/0.39** ^***^	0.18^***^/-0.05	0.06/-0.07	**0.39** ^***^ **/0.45** ^***^	1				
Perceived influence over health	**0.13** ^*^ **/0.25** ^***^	**0.16** ^**^ **/0.33** ^***^	0.05/-0.07	-0.05/-0.01	**0.17** ^**^ **/0.25** ^***^	**0.33** ^***^ **/0.46** ^***^	1			
PHQ-2	**0.18** ^***^ **/0.23** ^***^	**0.41** ^***^ **/0.34** ^***^	*0.10*^(*)^/-0.03	-0.04/-0.06	**0.54** ^***^ **/0.56** ^***^	**0.41** ^***^ **/0.45** ^***^	**0.16** ^**^ **/0.27** ^***^	1		
GAD-2	**0.25** ^***^ **/0.26** ^***^	**0.36** ^***^ **/0.32** ^***^	0.08/0.04	0.02/-0.05	**0.61** ^***^ **/0.68** ^***^	**0.38** ^***^ **/0.42** ^***^	**0.15** ^**^ **/0.27** ^***^	**0.66** ^***^ **/0.70** ^***^	1	
OSSS-3	**-0.18** ^***^ **/-0.15** ^**^	**-0.20** ^***^ **/-0.29** ^***^	0.01/0.05	0.04/*0.09*^(*)^	**-0.17** ^**^ **/-0.14** ^*^	**-0.17** ^**^ **/-0.16** ^**^	**-0.20** ^***^ **/-0.21** ^***^	**-0.24** ^***^ **/-0.21** ^***^	**-0.27** ^***^ **/-0.20** ^***^	1

Correlation analyses (Spearman’s Rho) for dental anxiety measured with the DAS, life satisfaction measured with L-1, annual dentist appointments, daily frequency of tooth brushing, perceived physical health measured with G-Score, perceived condition of health, perceived influence over health, indication for depression measured with PHQ-2, indication for anxiety measured with GAD-2, and social support measured with OSSS-3. Pearson’s r is reported. **Bold**: significant correlations, *italics*: trend-level significant correlations, ^***^ *p* < 0.001; ^**^ *p* < 0.01; ^*^ *p* < 0.05; ^(*)^ *p* < 0.10.

### Group comparisons

As assumptions of normality were not met, Wilcoxon signed-rank tests were performed to examine changes in dental anxiety over time across different participant groups based on oral health behaviors, demographic and social factors, and mental and physical health. For this purpose, only participants showing either a high expression of the respective predicting variable in both waves or a low expression of the respective variable in both waves were selected. DAS total scores were then compared for participants with either a high or a low expression of the respective variable across waves. Participants who brushed their teeth at least twice daily in wave 27 and wave 31 showed a significantly lower dental anxiety in wave 31 (8 [6–11]) than in wave 27 (9 [7–12]; z = -2.54, *p* = 0.006), whereas no significant change in the DAS score was observed among those brushing less frequently in wave 31 (8 (6.5–13.5) and wave 31 (8 [6–10]; z = -1.27, *p* = 0.102). Participants without an indication of depression in both waves (based on the PHQ-2) demonstrated a significant reduction in dental anxiety (wave 31: 8 [6–10]; wave 27: 9 [7–12]; z = -2.07, *p* = 0.019), whereas no significant change was found among those with an indication of depression in both waves (wave 31: 8 (6.5–13.5); wave 27: 8 [6–10]; z = -0.15, *p* = 0.441). Finally, participants reporting at least moderate levels of social support (measured with OSSS-3) in both waves exhibited a significant decrease in dental anxiety (wave 31: 8 [6–11]; wave 27: 9 [7–11]; z = -2.41, *p* = 0.008), while those with low social support in both waves showed no significant change (wave 31: 11 [8–14]; wave 27: 10 [8–13]; z = -0.37, *p* = 0.354). All additional Wilcoxon signed-rank tests, including median values, test statistics, and *p*-values, are summarized in Table 6.

**Table 6:** Group comparisons of DAS based on low and high expressions of the independent variables

Independent variable	Wave 27: 2013/2014 (median (IQR 25–75))	Wave 31: 2019/2020 (median (IQR 25–75))	z-value	*p*-value		*N*
Sex: male	8 (6–10)	8 (6–10)	-1.03	0.152	115	
Sex: female	10 (7–12)	9 (6–12)	-1.34	0.090	145	
Partner: yes	9 (6.5–11)	8 (6–11)	-1.34	0.090	145	
Partner: no	9 (7-12.5)	9 (7-12.5)	-0.31	0.379	33	
Children: yes	9 (6–11)	9 (6–11)	-1.54	0.066	217	
Children: no	9 (6–13)	8 (6–13)	-0.01	0.446	51	
L-1: high	8 (6–10)	8 (6–10)	-1.20	0.115	161	
L-1: low	11 (8–14)	11 (7-16.25)	-0.29	0.387	44	
Annual dentist appointments: min once a year	9 (6–11)	8 (6–11)	-1.00	0.160	110	
Annual dentist appointments: none a year	9 (7–12)	8 (7–12)	-0.01	0.495	99	
**Daily Frequency of teeth brushing: min twice a day**	**9 (7–12)**	**8 (6–11)**	**-2.54**	**0.006**	**223**	
Daily frequency of teeth brushing: max once a day	8 (6–10)	8 (6.5–13.5)	-1.27	0.102	33	
G-Score: healthy	8 (6–10)	8 (6–10)	-0.82	0.205	154	
G-Score: unhealthy	10 (7–14)	10 (6.5–13)	-1.48	0.069	69	
Perceived condition of health: healthy	8 (6–10)	8 (6–10)	-1.35	0.094	137	
Perceived condition of health: unhealthy	10 (8–15)	11 (7–14)	-1.06	0.143	63	
Perceived influence over health: high	8 (6–11)	8 (6–11)	-1.04	0.198	195	
Perceived influence over health: low	10 (8–12)	10 (7.5–12)	-0.35	0.365	37	
**PHQ-2: no indication for depression**	**8 (6–10)**	**7 (6–10)**	**-2.07**	**0.019**	**103**	
PHQ-2: indication for depression	10 (8–13)	10 (7–13)	-0.15	0.441	95	
GAD-2: no indication for anxiety	8 (6–10)	8 (6–10)	-0.68	0.250	87	
GAD-2: indication for anxiety	10 (7–13)	10 (7–13)	-0.44	0.664	109	
**OSSS-3: moderate to high social support**	**9 (7–11)**	**8 (6–11)**	**-2.41**	**0.008**	**217**	
OSSS-3: low social support	10 (8–13)	11 (8–14)	-0.37	0.354	19	

Descriptive values (median, iqr: inter quartile range, z-value, *p*-value, N) for group comparisons (Wilcoxon signed-rank tests) of dental anxiety measured with the DAS based on Sex (male/female), Age (years), Partners (yes/no), Life satisfaction (high/low), Annual dentist appointments (≥ 2/<2), Daily frequency of tooth brushing (≥ 2/<2), G-Score (healthy/unhealthy), Perceived condition of health (good/poor), Perceived influence over health (high/low), indication for depression measured with PHQ-2 (yes/no), indication for anxiety measured with GAD-2 (yes/no), and social support measured with OSSS-3 (moderate to high/low). **Bold**: significant group differences.

## Discussion

The present study is one of the few longitudinal investigations to examine the psychometric properties and correlates of the Dental Anxiety Scale (DAS) in a middle-aged population. Drawing on data from the Saxon Longitudinal Study (SLS), it offers new insights into the temporal stability of dental anxiety and its associations with psychosocial, behavioral, and health-related variables. Importantly, dental anxiety was assessed in non-dental interview settings, allowing the inclusion of individuals who may not be habitual users of oral health services and are often underrepresented in clinical research. Our findings underscore the enduring relevance of the DAS in this age group, while also identifying areas in which more differentiated or updated instruments could enhance assessment quality.

The excellent internal consistency and strong correlations between DAS total scores across measurement waves support the DAS’s reliability and stability in longitudinal research. The study’s extended follow-up period of seven years further strengthens the validity of these findings by capturing long-term trajectories. In line with previous research, our findings show a statistically significant decline in dental anxiety over time [29, 30, 67]. However, the clinical relevance of this change should be considered with caution, as dental anxiety remained relatively stable for most participants - an observation shown repeatedly [28, 29, 34]. At the same time, the data indicate that dental anxiety is not entirely static, as both increases and decreases were observed across categories, suggesting interindividual variation over time - a finding that has also been reported in previous studies by colleagues from Finland [28, 34]. Beyond measurement stability, the present study contributes to understanding potential determinants and modifiers of dental anxiety in midlife. Correlations with mental health indicators such as depression and anxiety symptoms, as well as with social support, life satisfaction, self-rated health, and perceived influence over health emphasize the complex psychosocial embedding of dental anxiety. Notably, participants who reported frequent tooth brushing, an absence of depressive symptoms, and higher levels of social support demonstrated the most pronounced reductions in dental anxiety over time. This may indicate that these factors function as protective mechanisms and point to the potential value of integrating behavioral and psychosocial support into oral health care, especially for vulnerable individuals. From a measurement perspective, the findings confirm key psychometric strengths of the DAS, including its one-factor structure replicated across waves, alongside indications of construct validity. This unidimensional format has been supported by previous research [1, 68] and offers several advantages. The typically brevity of such instruments reduces respondent burden and facilitates their use in both large-scale epidemiological studies and time-constrained clinical settings. However, such a structure might also represent a limitation. The DAS lacks sensitivity to distinguish between different components of dental anxiety, such as anticipatory and treatment-related fear, and does not include an item on fear of local anesthetic injections, as incorporated in more recent instruments like the MDAS [44]. Likewise, the IDAF-4 C offers a multidimensional, theory-driven framework encompassing emotional, cognitive, behavioral, and physiological aspects of dental anxiety [23]. Employing such instruments in future studies could provide a more nuanced understanding of dental anxiety and its manifestations.

Several limitations of this study must be acknowledged. As with most longitudinal research, issues of attrition and irregular follow-up can threaten data continuity and representativeness. In our sample, participation decreased over time, and not all individuals completed every wave. Since dropout reasons were not systematically recorded, the risk of attrition bias cannot be ruled out. Moreover, the study’s geographic restriction to East Germany may further limit the generalizability of results. While a comparison with a West German cohort would be desirable, no adequately analogous sample is available. Consequently, our findings primarily apply to individuals who grew up under the specific socio-political conditions of the former GDR. In terms of oral health behavior, the frequency of dental visits was self-reported and not matched with objective oral health indicators. Thus, it remains unclear whether reported visit patterns reflect appropriate use, overuse, or unmet need. Similarly, mental and physical health burdens were assessed using short forms of validated instruments. While efficient, these brief screeners may insufficiently capture complex constructs and introduce measurement bias. Indicators supporting the construct validity of the DAS include its consistent factorial structure across waves and the previously demonstrated convergent validity of the German version of the DAS through strong correlations with other established dental anxiety measures such as the DFS and IDAF-4 C [2, 69]. In addition, it shows only weak associations with constructs that are theoretically more distant, such as life satisfaction and social support in the present study, and oral health neglect in earlier research [70]. Nonetheless, further psychometric confirmation - such as confirmatory factor analyses - would be beneficial. The reliance on bivariate analyses limits control for confounding factors or interrelations between variables. Thus, future studies should consider applying multivariate models or structural equation modeling - to better reflect the complexity of associations and to explore potential causal pathways between dental anxiety and relevant predictors or outcomes. Another omission is the lack of sex- or gender-stratified analyses. Given that both dental anxiety and associated psychological symptoms are known to differ by sex and gender [16, 28, 32, 40], future research should address subgroup-specific patterns and vulnerabilities. Finally, it must be emphasized that, as an observational study, no causal conclusions can be drawn. To clarify causal mechanisms, future research should aim to include more diverse and representative samples, incorporate objective oral health assessments, and utilize interventional longitudinal designs that allow for stronger causal inference.

## Conclusions

Drawing on data from the Saxon Longitudinal Study (SLS), the present study provides robust evidence for the reliability, stability, and construct validity of the Dental Anxiety Scale (DAS) in a middle-aged German population. Although dental anxiety showed a statistically significant decline over time, its clinical relevance appears limited, suggesting that dental anxiety remains relatively stable for most individuals beyond midlife. Furthermore, the findings indicate a possible association between dental anxiety and psychosocial, behavioral, and health-related factors. This points to the potential value of interdisciplinary approaches that integrate psychological support into oral health care, particularly for individuals with elevated psychosocial burden. Although the DAS performs well psychometrically, its unidimensional structure limits the assessment of nuanced anxiety components. Future research could consider more differentiated instruments and examine subgroup-specific patterns, including gender differences to elucidate this matter.

## Data Availability

The data presented in this study are available upon request from the corresponding author. However, they are not publicly accessible due to privacy and confidentiality considerations.

## Abbreviations

DAS: Dental anxiety scale
DFS: Dental fear survey
GAD-2: Generalized anxiety disorder screener
G-Score: Health score
GDR: German democratic republic
IDAF-4C: The index of dental anxiety and fear
iqr: Inter quartile range
L-1: Short scale for measuring general life satisfaction
MDAS: Modified dental anxiety scale
OHB: Oral health behaviors
OSSS-3: Oslo social support scale
PCA: Principal component analyses
PHQ-4: Patient health questionnaire-4
SLS: Saxon longitudinal study
SPSS: Statistical software for the social science
STROBE: Strengthening the reporting of observational studies in epidemiology
